# A Case Report on Bismuth-Induced Encephalopathy Secondary to Pepto-Bismol

**DOI:** 10.7759/cureus.109840

**Published:** 2026-05-28

**Authors:** Nathaniel G Blanchard, Monica Sanjuan, Nathaniel Wang, Sheila C Lally

**Affiliations:** 1 Otolaryngology, Michigan State University College of Osteopathic Medicine, East Lansing, USA; 2 Internal Medicine, Michigan State University College of Osteopathic Medicine, East Lansing, USA; 3 General Surgery, Michigan State University College of Osteopathic Medicine, East Lansing, USA; 4 General Surgery, St. Michael Medical Center, Seattle, USA

**Keywords:** bismuth toxicity, colitis, drug toxicity, encephalopathy, parkinsonism

## Abstract

Bismuth subsalicylate is a widely available over-the-counter medication commonly used for gastrointestinal symptoms. Although generally considered safe, prolonged use can result in systemic toxicity, including severe neurological manifestations that are often underrecognized. We report the case of a 77-year-old man with microscopic colitis, lymphocytic subtype, who developed progressive encephalopathy, proximal muscle weakness, tremor, and gait instability after more than two years of daily bismuth subsalicylate use. His clinical course was marked by fluctuating neurological symptoms, multiple hospitalizations, extensive negative diagnostic evaluations, and an initial misdiagnosis of a Parkinsonian disorder. Serum testing ultimately revealed markedly elevated bismuth levels. Discontinuation of bismuth subsalicylate and supportive care resulted in gradual clinical improvement, with normalization of serum bismuth levels and gradual neurological recovery over several months. This case highlights the diagnostic challenges associated with bismuth-induced neurotoxicity and underscores the importance of thorough medication reconciliation, including over-the-counter agents. Increased clinician awareness is critical, as prompt recognition and discontinuation of bismuth-containing products can lead to substantial neurological recovery and prevent unnecessary diagnostic interventions.

## Introduction

Bismuth subsalicylate (BSS) (brand name: Pepto-Bismol®) was first introduced in the early 1900s as a treatment for cholera infections [1,2]. In 1939, the U.S. Food and Drug Administration approved BSS for the management of diarrhea, heartburn, indigestion, nausea, and general gastrointestinal upset [1]. In addition to its labeled indications, BSS is commonly used off-label for the treatment of *Helicobacter pylori* infection and traveler’s diarrhea [1]. The Norwich Pharmacal Company, the original manufacturer of BSS, promoted the medication as an “elegant, pleasantly flavored” mixture, contributing to its widespread acceptance for oral use [2]. Over the past century, BSS has remained a popular and readily available over-the-counter medication used in households throughout the United States to manage common gastrointestinal complaints.

BSS is well documented to cause mild adverse effects, including nausea, diarrhea, and black discoloration of stools [1]. These effects are generally considered benign and therefore rarely deter continued use. With increasing life expectancy and the widespread availability of Pepto-Bismol, prolonged use -- particularly among older adults -- has become more common and has contributed to a growing number of reported cases of BSS toxicity. Although rare, bismuth toxicity can present with a broad spectrum of neurological manifestations, including encephalopathy, proximal muscle weakness, myoclonus, tremor, psychiatric disturbances, and other neuropsychiatric symptoms [1,3]. Due to the rarity of this condition and the non-specific nature of its presentation, which frequently overlaps with more prevalent neurologic or systemic disorders, bismuth toxicity often goes unrecognized by both patients and healthcare professionals [3,4]. It is widely accepted that serum bismuth levels above 50 µg/L indicate severe intoxication and are typically associated with toxic neurological effects [5]. However, bismuth levels are not routinely measured in standard blood panels, as samples require quantification by mass spectrometry. As a result, diagnosis and treatment may be delayed, even when a comprehensive medication history is obtained [4].

In this report, we describe a 77-year-old patient with a prolonged history of BSS use who experienced progressive neurological symptoms for more than one year before bismuth toxicity was correctly identified. By detailing the clinical course, diagnostic challenges, and resolution following discontinuation of BSS, this case aims to increase awareness of bismuth-induced neurotoxicity among clinicians and the general public.

## Case presentation

The patient is a 77-year-old white male with a four-year history of BSS use (two tablets orally twice daily) for symptomatic management of lymphocytic colitis. He was initially diagnosed with colitis in June 2021 and was treated with budesonide for approximately two months. The budesonide was discontinued in August 2021, and BSS therapy was initiated. The patient reported taking six to eight tablets daily, though he later changed to two tablets in the morning and two at night after his symptoms stabilized. He presented to his primary care physician in December 2024 with intermittent and progressive neurological symptoms.

Symptom onset

The patient's symptoms began in March 2023 and consisted of subtle, intermittent neurological complaints. Initially, these symptoms occurred sporadically. At his annual physical examination in August of 2023, he was reportedly in excellent health. Around this time, he discontinued tamsulosin (Flomax) due to limited effects and an unpleasant taste, and his doctor switched him to mirabegron, which he took for nine days.

Approximately five days after his annual physical examination, the patient developed profound fatigue characterized by excessive somnolence and an inability to perform daily activities. He contacted his primary care physician and reported a significant decline in his health status. Believing the symptoms were medication-related, he discontinued mirabegron after nine days. Despite being off the medication for more than a week, his symptoms persisted. He described feeling extremely weak with cognitive changes and noted vivid dreams. He was advised that medication effects can take up to two weeks to resolve and to follow up if his symptoms do not improve.

Over the ensuing months, he underwent evaluations by multiple providers; no definitive diagnosis was reached. By November 2023, his condition had nearly returned to baseline, though he continued to experience mild proximal muscle weakness and generalized fatigue, particularly in the mornings.

Exacerbation of symptoms

In March 2024, the patient experienced a marked exacerbation of symptoms, including severe fatigue and balance disturbances, that significantly limited his daily activities. This acute decline was followed by a temporary improvement, creating a false sense of recovery. His bismuth intake remained unchanged throughout this period.

In August 2024, the patient contacted several physicians who had previously evaluated him, reporting that although his symptoms had recurred earlier in the year, he was currently feeling well, aside from persistent leg weakness. Laboratory studies obtained at that time demonstrated multiple minor, unrelated abnormalities. He requested guidance from his various providers and expressed concern that his symptoms might recur; he did not receive any recommendations.

In September 2024, the patient experienced another significant decline in functional status, representing the most severe exacerbation to date. He developed pronounced tremors, spasticity, and an inability to stand independently. During this period, he was involved in an airplane crash but fortunately sustained no traumatic injuries. Approximately one week later, he contacted his physicians to report worsening fatigue and progressive lower extremity weakness.

He subsequently presented to the emergency department, where he was diagnosed with an unspecified Parkinsonian disorder and was started on carbidopa-levodopa (Sinemet). His condition rapidly deteriorated, with worsening cognition, inability to ambulate, and episodes of agitation. He was hospitalized for one week and underwent extensive diagnostic testing, including laboratory studies, toxicology screening, lumbar puncture, CT imaging of the head, and MRI of the brain; all results were unremarkable.

Following hospitalization, the patient completed 13 days at an inpatient rehabilitation program before being discharged home with continued medical support. During rehabilitation, his cognition remained impaired, and he experienced severe fatigue. During this period, he was not taking BSS and was only receiving carbidopa-levodopa three times daily. His condition gradually improved, and he was discharged home, at which point he resumed taking Pepto-Bismol.

On his first night home, the patient sustained a fall, resulting in a burst fracture of his lower spine, further impairing his mobility. While his back pain improved modestly with rest and physical therapy, he experienced another neurological decline in December 2024, characterized by dysphagia and loss of bowel and bladder control. This culminated in a choking episode that prompted another emergency department visit.

Diagnosis and resolution of symptoms

**Figure 1:**
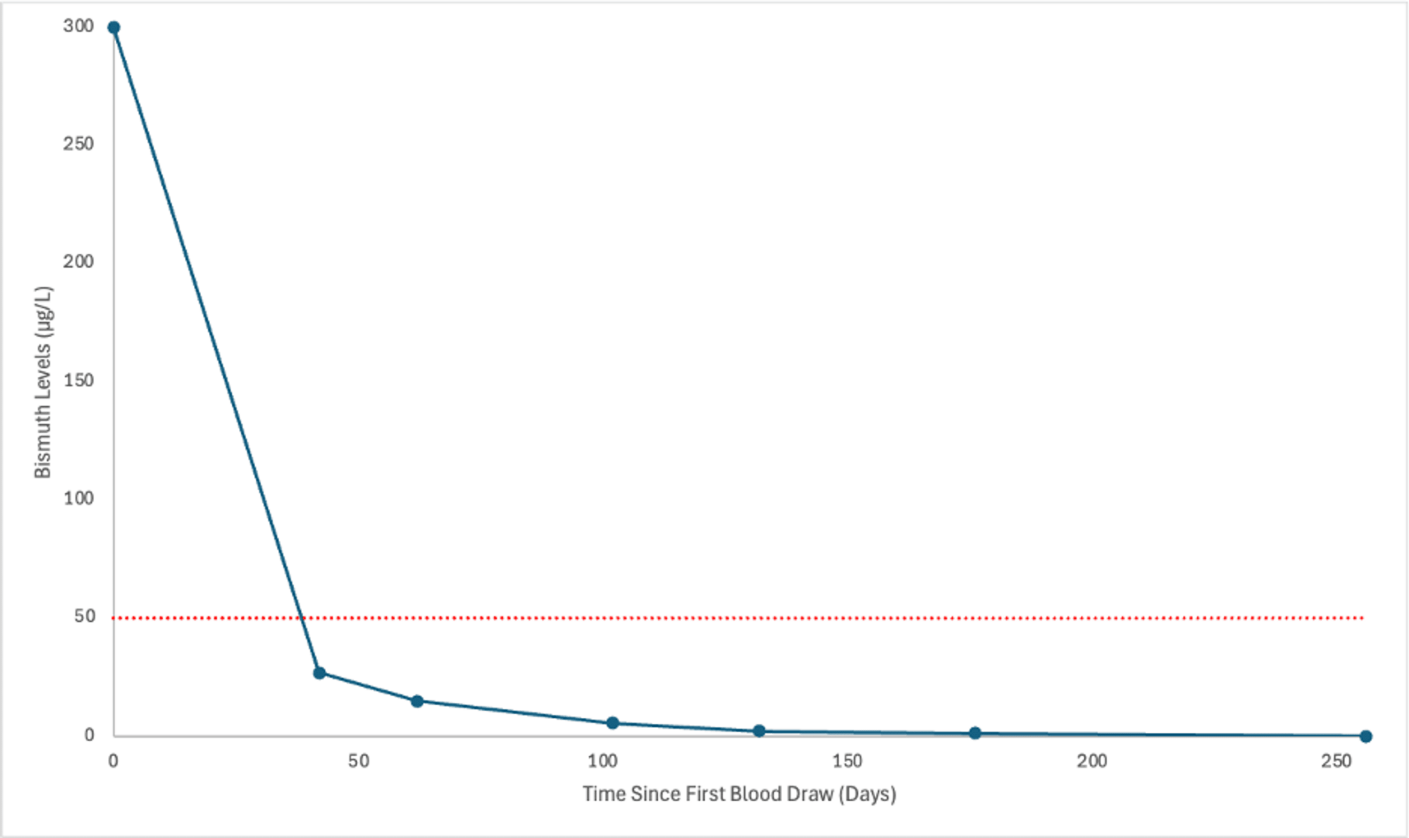
Serum bismuth levels (µg/L) Serum bismuth levels (µg/L) over a span of 256 days from initial (day 0) measurement of 300 µg/L. A red horizontal line at Y = 50 µg/L marks the upper limit of bismuth concentration in blood serum that results in toxic neurological effects.

During this emergency department evaluation, gastroenterology was consulted for assessment of dysphagia. Review of the patient’s medical history revealed prolonged and substantial use of BSS. Bismuth toxicity was suspected as the underlying cause of his chronic encephalopathy-like symptoms. Serum testing demonstrated a markedly elevated bismuth concentration of 300 µg/L (normal <5 µg/L; expected levels during therapeutic use range from 5 to 50 µg/L) (Figure 1).

The patient was advised to immediately discontinue Pepto-Bismol and was managed with supportive care, including physical, occupational, and speech therapy. Despite discontinuation of BSS, he experienced his most severe decline approximately one week later, developing bowel and bladder incontinence and requiring a walker for ambulation. In-home healthcare services were initiated.

Over the following weeks, the patient gradually improved, and home care was discontinued. Serial monitoring demonstrated progressive reductions in serum bismuth levels. Six weeks after discontinuation, his bismuth level had decreased to 27 µg/L, corresponding with his ongoing neurological improvement. His bismuth levels continued to decline, and no detectable bismuth was present nine months after cessation (Figure 1). This drop in bismuth level corresponded with his sustained clinical recovery.

## Discussion

Summary of key findings

This case illustrates the clinical course of bismuth-induced encephalopathy in a 77-year-old man following prolonged ingestion of BSS for management of lymphocytic colitis. Given the limited contemporary literature describing similar cases, this report contributes valuable insight into the progression, diagnostic challenges, and reversibility of bismuth neurotoxicity. Because the primary treatment is prompt discontinuation of bismuth-containing products, recognition of characteristic symptom patterns is critical [6]. Early identification may facilitate more rapid neurological recovery and prevent unnecessary diagnostic interventions.

Pathophysiological considerations

The mechanisms underlying the fluctuating and recurrent neurological exacerbations observed in this patient remain unclear. One plausible contributor is psychological stress and dysregulation of the brain-gut axis [1]. Notably, the patient experienced a significant neurological decline in December 2024 following a traumatic personal aircraft crash with associated psychological distress. Acute stress may alter gastrointestinal motility or permeability, potentially increasing systemic bismuth absorption [7]. Similar observations have been described by Borbinha et al., who reported symptom progression in a patient with chronic bismuth exposure following a major life stressor [3].

Additional contributors may include enhanced absorption related to gastrointestinal mucosal inflammation, altered gastric pH, or chronic colitis [8]. Polypharmacy and age-related declines in renal clearance may also have impaired bismuth excretion, facilitating accumulation and neurotoxicity [9,10]. Although these mechanisms cannot be definitively confirmed, they are consistent with established pharmacokinetic principles and prior case reports.

Diagnostic challenges and clinical significance

The differential diagnosis for encephalopathy is broad and includes neurodegenerative, infectious, autoimmune, metabolic, vascular, and toxic etiologies [11]. Bismuth-induced encephalopathy presents non-specifically and can mimic many of these conditions, underscoring the importance of a thorough and systematic evaluation [1]. This case highlights the need for meticulous medication reconciliation, including explicit inquiry into over-the-counter and self-administered medications. Because bismuth neurotoxicity is often reversible, timely recognition and discontinuation of bismuth are essential to improving outcomes.

Public health and prescribing implications

This case underscores the potential for serious neurological complications from over-the-counter medications widely perceived as safe. Prolonged or excessive use of bismuth-containing products can result in significant toxicity, particularly in older adults [9,10]. Several European countries have restricted or banned certain bismuth compounds due to similar adverse effects, reflecting international concern regarding their safety [12]. Clinicians should routinely inquire about non-prescription medication use, counsel patients on appropriate dosing and duration, and monitor individuals who rely on chronic symptomatic therapies. 

Increased awareness of bismuth-induced encephalopathy may prevent progression to severe neurological impairment and improve patient outcomes. Ultimately, this report reinforces the importance of vigilance in evaluating over-the-counter medication use as part of comprehensive clinical care.

## Conclusions

Bismuth-induced neurotoxicity is a rare but clinically important adverse effect of a widely available over-the-counter medication. Its presentation is often non-specific and may mimic more common neurologic or systemic conditions, frequently resulting in delayed diagnosis despite extensive evaluation. This case highlights the importance of maintaining a high index of suspicion for bismuth toxicity in patients presenting with unexplained encephalopathy or progressive neurologic symptoms, particularly in older adults and those with prolonged or high-dose exposure to BSS. Thorough medication reconciliation, including careful review of non-prescription agents, is essential for timely recognition and diagnosis. Although further research is needed to better characterize the underlying pathophysiology and spectrum of clinical presentation, prompt discontinuation of bismuth-containing products remains the cornerstone of management. Early identification and cessation of exposure are associated with significant neurologic recovery, underscoring both the clinical importance and reversibility of this condition.
